# Understanding the Molecular Mechanisms of Succinic Semialdehyde Dehydrogenase Deficiency (SSADHD): Towards the Development of SSADH-Targeted Medicine

**DOI:** 10.3390/ijms23052606

**Published:** 2022-02-26

**Authors:** Henry H. C. Lee, Gabrielle E. McGinty, Phillip L. Pearl, Alexander Rotenberg

**Affiliations:** 1 F.M. Kirby Neurobiology Center, Boston Children’s Hospital, Boston, MA 02115, USA; gabrielle.mcginty@childrens.harvard.edu (G.E.M.); alexander.rotenberg@childrens.harvard.edu (A.R.); 2 Rosamund Stone Zander Translational Neuroscience Center, Boston Children’s Hospital, Boston, MA 02115, USA; 3 Division of Epilepsy & Clinical Neurophysiology, Department of Neurology, Boston Children’s Hospital, Boston, MA 02115, USA; phillip.pearl@childrens.harvard.edu

**Keywords:** succinic semialdehyde dehydrogenase deficiency (SSADHD), *ALDH5A1*, γ-aminobutyric acid (GABA), γ-hydroxybutyrate (GHB), epilepsy, inhibition, GABA receptors, plasticity, mouse model, gene therapy, enzyme replacement therapy

## Abstract

Succinic semialdehyde dehydrogenase deficiency (SSADHD) is a rare genetic disorder caused by inefficient metabolic breakdown of the major inhibitory neurotransmitter, γ-aminobutyric acid (GABA). Pathologic brain accumulation of GABA and γ-hydroxybutyrate (GHB), a neuroactive by-product of GABA catabolism, leads to a multitude of molecular abnormalities beginning in early life, culminating in multifaceted clinical presentations including delayed psychomotor development, intellectual disability, hypotonia, and ataxia. Paradoxically, over half of patients with SSADHD also develop epilepsy and face a significant risk of sudden unexpected death in epilepsy (SUDEP). Here, we review some of the relevant molecular mechanisms through which impaired synaptic inhibition, astrocytic malfunctions and myelin defects might contribute to the complex SSADHD phenotype. We also discuss the gaps in knowledge that need to be addressed for the implementation of successful gene and enzyme replacement SSADHD therapies. We conclude with a description of a novel SSADHD mouse model that enables ‘on-demand’ SSADH restoration, allowing proof-of-concept studies to fine-tune SSADH restoration in preparation for eventual human trials.

## 1. Introduction

Succinic semialdehyde dehydrogenase deficiency (SSADHD) is a rare genetic metabolic disorder caused by loss-of-function mutations of the *ALDH5A1* gene. *ALDH5A1* encodes SSADH which is essential for the mitochondrial breakdown of succinic semialdehyde (SSA), a γ-aminobutyric acid (GABA) downstream metabolite, into succinate. In the absence of SSADH, SSA conversion to succinate is prohibited, leading to SSA conversion to γ-hydroxybutyrate (GHB). Both GABA and GHB are accumulated in the brain and body fluids (cerebrospinal fluid, blood) up to pathologic levels, resulting in numerous downstream neurological and metabolic abnormalities.

**Figure 1:**
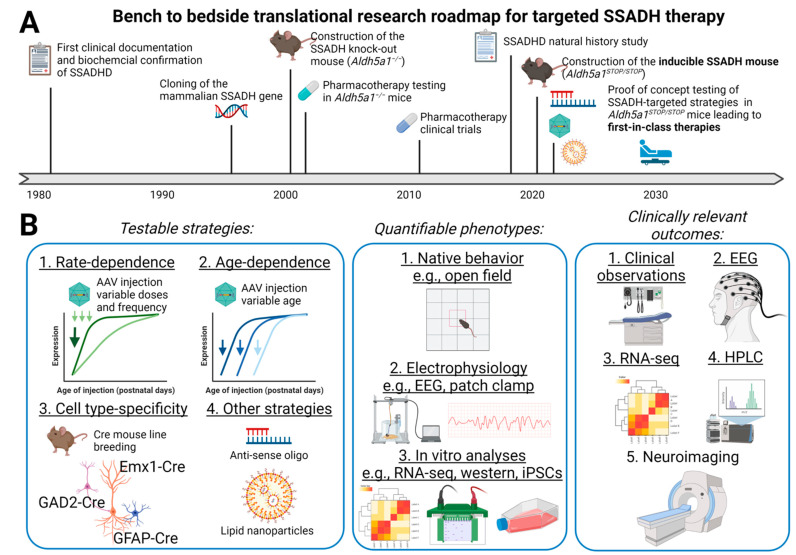
Translational roadmap of bench to bedside discovery and development of SSADH-targeted medicine. (**A**) A brief timeline of SSADH research from initial discovery to potential first-in-class SSADH-restoring therapies development. Originally documented and confirmed biochemically in the early 1980’s, there are important landmarks including cloning of the *ALDH5A1* gene, construction of the first mouse model *aldh5a1*^−/−^, quickly followed by a barrage of pharmacotherapy testing from preclinical to clinical settings. The recent launch of the SSADHD Natural History Study and the ongoing construction work of the novel SSADHD mouse model *aldh5a1^STOP/STOP^* are instrumental to accelerate bench to bedside translational research for SSADHD. (**B**) SSADH-restoring strategies are readily testable using the *aldh5a1^STOP/STOP^* mice. Key strategies include: (1) rate-dependence, (2) age-dependence, and (3) cell type-specificity. In addition, other novel strategies such as anti-sense oligonucleotide or lipid nanoparticles delivery approach can be tested. The *aldh5a1^STOP/STOP^* will be an in vivo platform allowing quantifiable assessment of phenotype reversal, from behavior to the molecular level. Relevant preclinical results will combine with the Natural History Study to form the basis for prognostic assessment in eventual clinical trials.

To date, SSADHD treatments remain symptomatic and only minimally effective. A fundamentally different approach is targeted SSADH restoration, which may be accomplished via gene therapy or enzyme replacement therapy (ERT). While gene and enzyme replacement therapies are still in their infancy, we envision that the current technology and research environment enable a quick transition from basic bench side research to clinical trials (Figure 1A). We therefore describe our ongoing work aimed to develop SSADH-targeted treatments. By generating a novel SSADHD mouse model which allows ‘on-demand’ SSADH restoration, we aim to understand the impact of SSADH restoration from molecular level to animal behaviors. The ongoing SSADHD Natural History Study provides correlating clinical data for relevant biomarkers development. These combined efforts will accelerate the transition from bench to bedside development of the eventual safe and effective therapeutic products.

## 2. A Brief History and Update on SSADHD Research

Identified by Jacobs and colleagues in the early eighties of the last century, SSADHD was first described as a rare form of inborn error of metabolism characterized by gamma-hydroxybutyric aciduria coupled with neurological abnormalities [1]. Gibson and colleagues subsequently discovered the biochemical underpinnings of SSADH enzyme deficiency in patients, defining this disorder in the medical literature [2]. The biochemical hallmark of SSADHD is a pathologic build-up of GABA and GHB in the brain and body fluids, due to impaired SSADH enzymatic activities necessary for GABA catabolism traceable to embryonic stages [3]. Electron microscopic imaging of the rat cerebellar Purkinje neurons and liver hepatocytes revealed the mitochondrial localization of SSADH [4]. Molecular cloning in the mid-nineties enabled genomic mapping and sequencing of the mammalian SSADH-encoding gene, *ALDH5A1* [5], revealing a range of loss of function mutations in *ALDH5A1* associated with this disorder documented in the following decades [5,6,7,8,9,10,11].

Clinically, SSADHD is characterized by a complex phenotype that includes neurodevelopmental delay, intellectual disability, movement disorders, and epilepsy. Notably, patients with SSADHD are at risk for sudden unexpected death in epilepsy (SUDEP) [12,13,14,15,16,17,18,19]. Due to the diverse symptoms shared among other common neurodevelopmental disorders, diagnosis of SSADHD is critically dependent on combined genetic (i.e., *ALDH5A1* sequencing) and metabolic testing [20]. Conventional treatment for SSADHD relies on symptomatic relief (mainly in the form of pharmacologic seizure control [21,22,23,24]) and behavioral therapies customized toward specific symptoms and patient needs (movement disorders, speech delay, etc.) [13,21,24,25,26]. However, therapies that target the underlying enzyme deficiency in the form of gene therapy or ERT are non-existent to date.

The currently available mouse model of SSADHD, the *aldh5a1* knock-out mice, *aldh5a1*^−/−^, mimics a severe form of the human disorder [27]. *Aldh5a1*^−/−^ mice develop spontaneous, absence seizures at two weeks of postnatal age, progressing to generalized tonic–clonic seizures and eventual lethality at an age of about three weeks [28,29,30]. This mouse model has been instrumental for testing a range of pharmacologic and metabolic rescue approaches, including taurine [27,31], GABA transaminase (GABA-T) irreversible inhibitor vigabatrin [27,31], GABA_B_ receptor antagonists [27,28], GHB receptor antagonist [31,32], mechanistic target of rapamycin (mTOR) inhibitors [33,34] and ketogenic diet [35,36]. These strategies were based on the hypothesis that targetable components in the GABA catabolic pathway or pertinent metabolic signaling cascades are potential therapeutic targets. Importantly, these treatment strategies produced appreciable therapeutic outcomes in *aldh5a1*^−/−^ mice including seizure suppression, phenotypic reversal and extended lifespan. These promising preclinical results also formed the scientific basis for clinical drug trials, including taurine [37,38] and a recently completed study of GABA_B_R antagonist SGS-742 [39]. Yet, while these drugs were well-tolerated in patients, their therapeutic effects were rather limited.

A fundamentally different alternative therapeutic approach for SSADHD treatment involves the direct targeting of the underlying deficiency instead of symptomatic relief or GABAergic antagonism. SSADH-targeted medicine can exist in the form of: (1) gene therapy to amend the deficient *ALDH5A1* gene, and (2) ERT to supplement impaired SSADH catalytic activities. Both therapies are currently unavailable to patients, but proof-of-concept studies in *aldh5a1*^−/−^ mice showed striking therapeutic efficacy. Using a liver-directed adenoviral approach, Gupta and colleagues demonstrated increased *aldh5a1*^−/−^ mice survival and brain metabolite reversal [40]. Recently, Vogel and colleagues demonstrated that systemic injection of recombinant SSADH proteins also enhanced *aldh5a1*^−/−^ mice survival accompanied by corrected molecular and metabolite measures in the brain [41]. These encouraging results indicate that SSADH-targeted approaches have appreciable potential for treating SSADHD symptoms. Given the recent technology advancement in gene therapy [42,43,44] and ERT [45,46], these novel approaches are forthcoming viable therapeutic options for SSADHD patients.

In ongoing work, we aim to apply first principles reasoning in developing SSADH-targeted medicine. First, we begin with understanding the fundamental pathophysiology of the disorder (i.e., *ALDH5A1* malfunctions) and its immediate impacts from the systems level down to the molecular level. This is achieved at two parallel fronts: (1) the SSADHD Natural History Study defining the natural course and identify disease correlating biomarkers [47]. This provides a unique opportunity to study relevant pathophysiology via electrophysiologic (electroencephalography, EEG), neuroimaging (magnetic resonance imaging, MRI), and metabolite analyses in a longitudinally manner. (2) Development of a clinically relevant disease mouse model allowing ‘deep phenotype’ characterization of the mouse from behaviors down to the molecular level (see details below) to mirror the patient’s findings. In some situations, new findings from mouse data might provide insights updating clinical research directions. Second, we break down these complex clinical observations and molecular findings into basic elements (e.g., EEG abnormalities, metabolic signatures, gene expression changes) and test in the mouse model whether direct repairing the *ALDH5A1* gene (as in gene therapy) or supplementing SSADH (as in ERT) might reverse some (if not all) of these abnormalities to the normal level. Fine-tuning of SSADH-targeted strategies using a mouse model will be critical to avoid potential adverse side effects and achieve the best efficient rescue outcomes. Third, favorable results from the preclinical research will then serve as a strong basis for developing actual therapeutic products testable in eventual clinical trials (Figure 1B).

As a critical step toward developing SSADH-targeted medicine, we are constructing a clinically relevant novel SSADHD mouse model which allows ‘on-demand’ SSADH restoration to test key SSADH-restoring parameters. In principle, this mouse has no SSADH at baseline due to the inactivation of the *aldh5a1* gene, mimicking the human disorder. However, a genetic switch is engineered so that it allows the inactivated *aldh5a1* gene to turn back on in a highly controllable manner [48]. This novel mouse model will allow investigators to understand how SSADH restoration impacts the brain from the molecular level all the way up to the behavioral level via a ‘deep phenotyping’ repertoire.

In the following sections, we discuss some of the molecular signaling and pathways most relevant for seizures in SSADHD. These molecular changes observable in the disease animal model are the first set of metrics to test safety and efficacy of SSADH-targeted medicine.

## 3. GABA, Chloride Homeostasis and GHB Signaling in Neuronal Inhibitory Control

**Figure 2:**
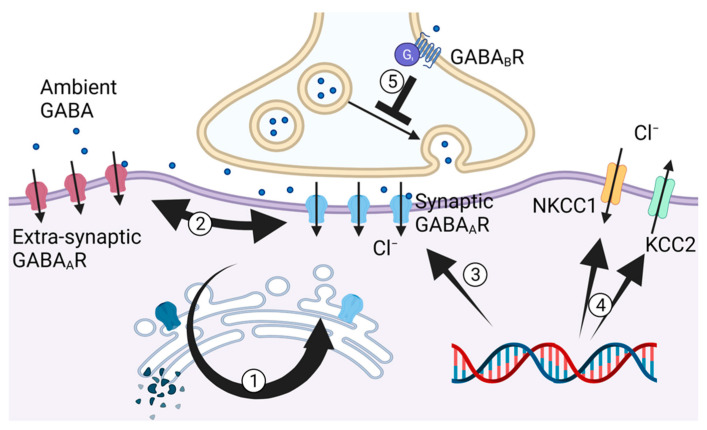
Regulatory mechanisms of GABAergic receptors, sodium potassium chloride co-transporter NKCC1, and potassium chloride co-transporter KCC2. Intracellular trafficking of GABA_A_R involves internalization into the Golgi, lysosomal degradation, or recycling through the endoplasmic reticulum and membrane re-insertion (1). Lateral mobilization of GABA_A_R between synaptic and extrasynaptic domains (2) and transcriptional regulation of GABA_A_R (3). Transcriptional regulation of NKCC1 and KCC2 (4). Presynaptic GABA_B_R regulating presynaptic neurotransmitter release (5).

GABA is the principal inhibitory neurotransmitter across the brain, essential for signal processing [49], neural plasticity [50] and excitatory control [51]. GABAergic neurotransmission depends on the synaptic release of GABA from presynaptic GABAergic terminals and its binding to ionotropic GABA_A_ receptors [52] and metabotropic GABA_B_ receptors [53] in postsynaptic domains (Figure 2). Both GABA release and receptor representations on neuronal surface are strictly regulated [54,55]. For example, intracellular trafficking and recycling of GABA_A_ receptors between the endoplasmic reticulum and neuronal surface are subunit-specific and dependent on phosphorylation status and molecular cargo–adaptor assembly [54]. GABA_A_ receptors are classified into synaptic and extrasynaptic populations that have subunit-specific composition and signatures [56]. Their lateral mobility between synaptic and extrasynaptic GABA_A_ receptors are strictly controlled. Ligand binding desensitizing the receptor may also contribute to long lasting potentiation mechanisms [57]. Transcriptional events for GABA_A_ receptors are also modifiable via signaling cross-talks of transcriptional sensors in a subunit-specific manner [58]. At the presynaptic domain, GABA_B_ receptors play a key role in vesicular neurotransmitter release. These heterodimeric G-protein coupled receptors are highly regulated and provide more sustained effects in modulating synaptic excitatory and inhibitory actions [59]. Disruption or malfunction of these processes are the basis of a wide range of neurologic disorders [60,61].

Relevant for SSADHD, pathologic GABA accumulation is a biochemical hallmark detectable in patients’ body fluid samples [62], which has been recently verified using state-of-the-art neuroimaging techniques [63]. At a functional level, GABA accumulation is reflected by longer cortical silent periods in patients using transcranial magnetic stimulation (TMS) measures [64]. Persistent levels of GABA lead to use-dependent down-regulation of GABA_A_ receptors revealed in neuroimaging data from SSADHD patients [65] and confirmed by molecular analysis in *aldh5a1*^−/−^ mice [33]. Down-regulation of GABA_A_ receptors has also been verified recently in postmortem patient brain tissues via gene expression analyses [19]. The profound compensatory down-regulation of GABA_A_ receptors might contribute to the overall reduced inhibitory tone ultimately leading to seizures in patients, leading to the hypothesis that too rapid SSADH restoration without compensatory GABA_A_ receptor up-regulation might inadvertently lead to a relatively hypo-GABAergic state which induces further seizures and brain damage [48].

In SSADHD patients, GABA accumulation in brain follows a downward trajectory across development [62], suggesting a more drastic excitatory/inhibitory (E/I) imbalance in early life. The developmental pathological aspect of SSADHD has been implicated in the literature [18,62], and is now being further pursued in the ongoing SSADHD Natural History Study. This developmental aspect carries significant implications when developing successful SSADH-targeted medicine, i.e., whether a critical period exists for symptom reversal.

A fundamental key determinant of fast inhibitory actions mediated by GABA_A_ receptors is the transmembrane chloride gradient maintained by potassium chloride co-transporter KCC2 and sodium potassium co-transporter NKCC1 [66] (Figure 2). In mature neurons, GABA_A_ receptor-mediated inward chloride movement results in membrane hyperpolarization and neuronal inhibition. This polarity, however, might be reversed upon downregulation of the KCC2 exporter [67]. KCC2 is highly regulated at a transcriptional and post-translational level. Indeed, in *aldh5a1*^−/−^ mice, NKCC1 up-regulation is higher than that of KCC2 at a transcriptional level [41]. This might lead to an overall chloride influx and a depolarizing or excitatory GABA-mediated current reminiscent of an immature brain.

**Figure 3:**
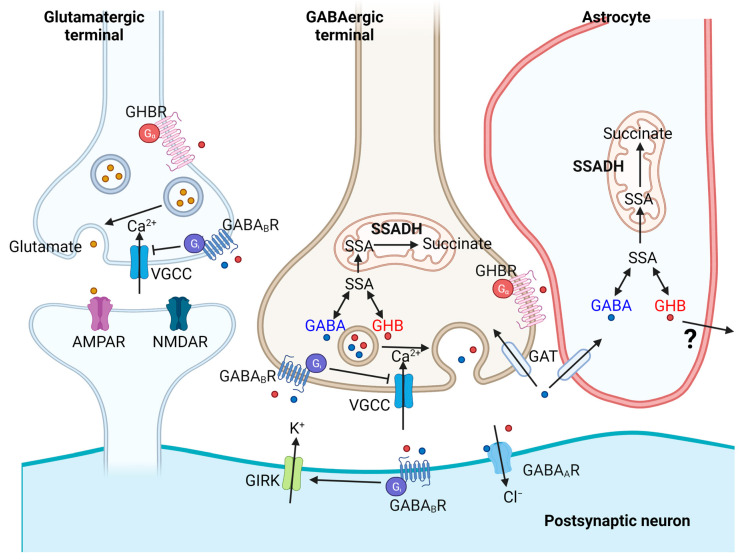
Schematic diagram showing key synaptic mechanisms relevant for SSADHD. SSADH is a mitochondrial enzyme essential for the conversion of SSA (succinic semialdehyde) to succinate downstream of γ-aminobutyric acid (GABA) catabolism. In SSADHD, presynaptic release of GABA and metabolic by-product γ-hydroxybutyrate (GHB) from GABAergic terminals activate type-A and type-B GABA receptors (GABA_A_R, GABA_B_R) and GHB receptors (GHBR). GABA_A_R is an ionotropic chloride channel, which mediates postsynaptic hyperpolarization. GABA_B_R is a metabotropic G-protein coupled receptor, which mediates presynaptic blockade of voltage-gated calcium channels (VGCC) for synaptic vesicle release, and postsynaptic activation of G-protein-coupled inwardly rectifying potassium (GIRK) channels. Recycling of GABA into GABAergic terminals and astrocytes is mediated through the specific GABA transporter (GAT). G-protein coupled GHBRs are involved in both glutamatergic and GABAergic neurotransmission.

Besides GABA, the central pathology of SSADHD is the accumulation of GHB (Figure 3). GHB is broadly known as a central nervous system depressant with euphoric and relaxant effects, often associated with substance abuse [68]. Despite dose-dependent side effects, GHB has therapeutic value in treating narcolepsy [69]. Importantly, GHB is also an endogenous neuroactive GABA metabolite normally found in micromolar (µM) quantities across the brain [70]. In SSADHD, mitochondrial SSA conversion to succinate is aborted, leading to excessive GHB biosynthesis and accumulation in the brain and body fluids including cerebrospinal fluid and blood to over hundred-fold levels compared to normal individuals [62,71]. Overdriven GHB signaling is therefore likely one of the major factors in SSADH pathophysiology. Nevertheless, GHB-mediated intracellular signaling cascades (see section below) remain one of major research topics in understanding the molecular mechanisms underlying SSADHD pathophysiology. Interestingly, blood GHB levels decline with patient age [62], suggesting that compensatory metabolic mechanisms might be in place to lower GHB contents across development. Life-long neuropsychiatric manifestations in SSADHD might be triggered by early life GHB impact. In *aldh5a1*^−/−^ mice, high GHB levels are found in physiologic fluids (urine) and tissue homogenates (brain, liver) [27]. This also forms the basis of testing GHB receptor inhibitors in *aldh5a1*^−/−^ mice [31,32].

The neuroactive properties of GHB are attributed to its high affinity binding (activation in nM-µM range) to the GHB receptors [72], ionotropic α4β1δ-containing GABA_A_ receptors [73], and low affinity binding (activation in mM range) to G-protein coupled GABA_B_ receptors [74] and ionotropic α4β2/3δ-containing GABA_A_ receptors [75]. The intracellular signaling pathways mediated by GHB are likely to be diverse and are still under active investigation. GHB receptors are presynaptic G-protein coupled receptors whose activation leads to brain region-specific (cortex and hippocampus) decrease in adenylyl cyclase production [76]. In the rat thalamic ventral–basal nucleus, basal and K^+^-evoked extracellular GABA release is inhibited by GHB, reminiscent of absence-like seizures [77]. GHB also inhibits dopamine release in striatum of behaving rats, while anesthetic pretreatment completely abolished this effect [78,79], suggesting that certain GHB-mediated signaling effects are activity-dependent. Altogether, GHB exerts a wide range of neuromodulatory effects via neurotransmitter release blockade and binding to multiple neurotransmitter receptor systems. Relevant for SSADHD, *aldh5a1*^−/−^ mice survival was significantly improved by the application of GHB receptor antagonist NCS-382 [31] or GABA_B_ receptor antagonist CGP 35348 [27,31], thus underscoring the GHB contribution to SSADHD pathophysiology.

## 4. Astrocyte and Oligodendrocyte Dysfunction in SSADHD

**Figure 4:**
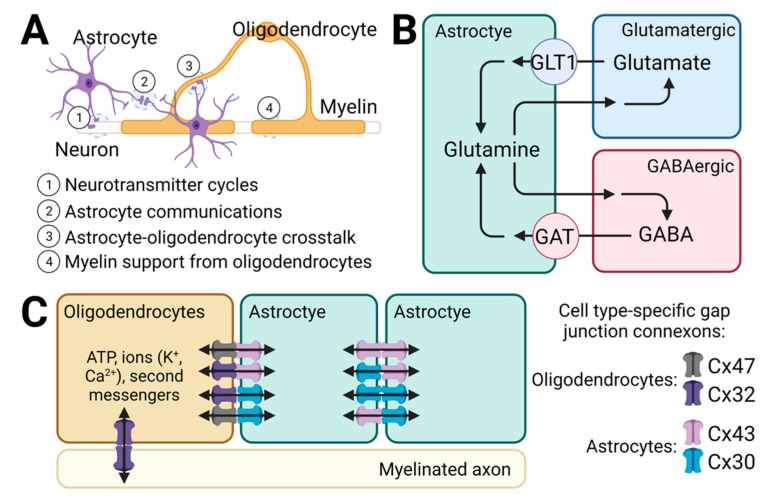
Astroglial-mediated mechanisms in neural cell communication. (**A**) Glial cells mediate a wide range of neurophysiological processes: (1) neurotransmitter cycles between astrocytes and neurons, (2) astrocyte communication, (3) crosstalk between astrocytes and oligodendrocytes, (4) oligodendrocyte-derived myelin enwrapping neurons. (**B**) Schematic diagram showing the neurotransmitter cycles. Excitatory (glutamate) and inhibitory (GABA) neurotransmitters reuptake into astrocyte via the glutamate transporter (GLT1) and the GABA transporter (GAT), respectively. Both neurotransmitters are metabolized to glutamine and are recycled back to glutamatergic and GABAergic neurons for the synthesis of their respective neurotransmitters. In SSADHD, pathologic accumulation of GABA likely has a direct impact on astrocyte recycling, contributing to astrocyte dysfunction. Glutamine reduction is observed in SSADHD patients and in the mouse model. (**C**) Astroglial communications are mediated by gap junctions which are made of cell type-specific connexons. Gap junctions mediate rapid exchange of small molecules including ATP, ions, and second messengers. Astrocyte dysfunction in SSADHD might manifest into a wide range of pathophysiological processes involving the vast astrocyte–oligodendrocyte–neuronal network amplified by gap junction abnormalities.

Astroglia are the most abundant cell types found in the brain, which are involved in a wide range of physiological processes (Figure 4A). Astrocytes are located at synapses regulating neural signal transmission and synapse development [80]. Astrocytes express glutamate transporter GLT1 important for glutamate homeostasis [81], as well as astrocyte-specific GABA transporter GAT-3 for GABA recycling [82] (Figure 4B). Astrocytes also regulate axonal growth via direct cellular contacts or indirect extracellular factor communication [83]. Furthermore, astrocytes are constantly surveying the brain endothelium regulating blood–brain barrier (BBB) functions [84]. Interestingly, GABA signaling likely also plays a role in regulating BBB functions relevant for epilepsy [85]. Upon brain injury, reactive astrogliosis is a key step to restore brain homeostasis and energy balance. However, in certain pathologic conditions where astrogliosis persists, further brain damage may result in epileptogenesis [86,87]. Astrocytes are also abundantly coupled through gap junctions ideal for the distribution of ions, adenosine triphosphates (ATPs), and second messengers [88,89] (Figure 4C). Gap junctions are made of cell-specific connexons (hemichannels) for cell–cell communications [90]. It has been shown that gap junctions contribute significantly to K^+^ buffering [91] and glutamate translocation [92], both of which are involved in E/I balance and are compromised in astrogliosis. In certain situations, non-junctional hemichannels might play a role in extracellular space communication [93].

Oligodendrocyte-mediated myelin also plays a key role in glial communication and support in the central nervous system [94]. Myelin supports the function of fast-spiking interneurons, maintaining E/I balance in the local circuits [95]. Accordingly, myelin defects and oligodendrocyte de-maturation is found in epileptic foci of patients with intractable epilepsy [96]. Interestingly, myelin also forms connexon-specific gap junctions with axons [97].

Relevant for SSADHD, the *ALDH5A1* gene is expressed across glial populations including astrocytes and oligodendrocytes [48]. Given the significant roles of glial populations in neurotransmitter cycles, cell–cell communication and myelin support, glial dysfunction is heavily implicated in SSADHD [98]. Biolipid myelin defects relevant for oligodendrocytes were observed in *aldh5a1*^−/−^ mice [99,100]. Recently, myelin-related phospholipid reduction and astrogliosis were confirmed in postmortem analyses in an SSADHD patient [19]. These findings correspond to impaired K^+^ and glutamate buffering (see above). In addition, astrocytes are responsible for neurotransmitter reuptake and bioconversion of glutamine. Correspondingly, low glutamine levels were observed in patients [16] and in *aldh5a1*^−/−^ mice [101], suggesting profound astrocyte dysfunction in SSADHD.

## 5. The Novel SSADHD Mouse Model, *aldh5a1^STOP/STOP^*

**Figure 5:**
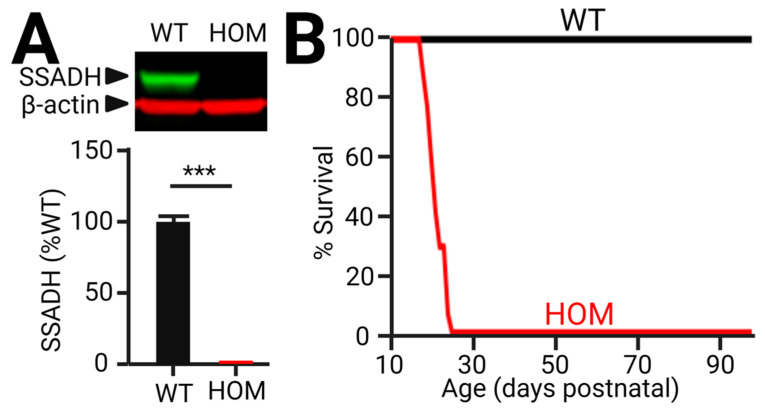
Cortical expression of SSADH and survivability of *aldh5a1^STOP/STOP^* mice. (**A**) Western blot analyses of cortical lysates from wild-type (WT) and homozygous mutant (HOM) *aldh5a1^STOP/STOP^* mice at postnatal age of 16 days. β-actin serves as protein loading control. Quantification of SSADH expression is expressed as % WT, showing HOM mice have virtually no SSADH expression. Values = Mean ± SEM. *** *p* < 0.001, unpaired *t*-test, n = 10 WT, 8 HOM mice. Both male and female mice were used. (**B**) Survivability of WT and HOM mice across development. Note a sharp plunge in HOM survival rate that occurs around postnatal age of 20–25 days, while no lethality was observed in WT. n = 30 WT, 17 HOM mice.

We previously described the concept of developing a novel SSADHD mouse model that allows ‘on-demand’ SSADH restoration [48]. In this design, we genetically engineer a gene cassette inactivating the endogenous *aldh5a1* gene in mice, mimicking the human SSADHD disorder. We anticipate that, at baseline, these mice (namely *aldh5a1^STOP/STOP^*) phenocopy *aldh5a1*^−/−^ exhibiting underdevelopment, spontaneous seizures, and premature death in early life [27]. In the presence of Cre recombinase, however, we anticipated that the inactivated *aldh5a1* gene will be restored and regulated under its own endogenous transcriptional control elements. Therefore, by controlling the way in which Cre is introduced into the *aldh5a1^STOP/STOP^* mice, we will be able to systematically investigate key SSADH restoration parameters. Indeed, pilot results indicated that *aldh5a1^STOP/STOP^* mice do not express SSADH in cortex, and they exhibit obligatory premature lethality before three weeks of postnatal age (Figure 5). These results suggest that our lox-STOP insertion leads to compulsory premature *aldh5a1* termination in *aldh5a1^STOP/STOP^* mice. The molecular strategy used in the *aldh5a1^STOP/STOP^* mouse is fundamentally different from that used in the *aldh5a1*^−/−^ mouse (i.e., *aldh5a1* exon 7 deletion), but these mice phenocopy each other in terms of seizure generation and premature lethality, highlighting the critical role of SSADH in neural development. Further molecular and behavioral characterization of *aldh5a1^STOP/STOP^* mice is ongoing. We will also test how *aldh5a1* restoration might lead to phenotypic rescue in these mice.

We hypothesize that given the profound use-dependent, compensatory down-regulation of GABA receptors, too rapid SSADH restoration without allowing for sufficient compensatory reversal of GABA receptor expression will lead to seizures and further brain damage and even death. In a proof-of-concept study using an adeno-associated virus encapsulated with blood–brain barrier penetrating capsid (AAV-PHP.eB), we demonstrated rate-dependent transgene expression via a differential dosing paradigm [48]. Here, we will use AAV-PHP.eB-Cre to achieve brain-wide Cre recombination and *aldh5a1* restoration in *aldh5a1^STOP/STOP^* mice. We will check survival rates, metabolite levels (GABA and GHB), protein expression (SSADH enzymatic activity, GABA_A_ receptors), electrographic seizures (using EEG) and video monitoring of native exploratory behaviors to ‘deep phenotype’ mice before and after treatment. Besides rate-dependent AAV-mediated rescue, we will also investigate the age at which AAV injection will lead to the best therapeutic outcome. The same phenotypic metrics will be used. Another relevant SSADH restoration parameter is cell-specificity. By crossing *aldh5a1^STOP/STOP^* with various conditional Cre-expressing lines, we will understand specific cell populations necessary for successful phenotypic rescue. *Aldh5a1* is broadly expressed in the brain, but its expression profile among various cell types is non-uniform [48]. For example, cell types including interneurons, certain populations of hippocampal pyramidal cells, astrocytes, and oligodendrocytes have higher *aldh5a1* expression, while microglia and pericytes have very limited *aldh5a1* expression (if at all). Conditional Cre lines targeting different cell populations such as Gad2-Cre (inhibitory interneuron-only) [102], Emx1-Cre (excitatory neuron-only) [103] and GFAP-Cre (astrocyte-only) [104], will be readily useful to dissect essential cell types for phenotype rescue. Results will be critical for future development of AAV vectors that require incorporating cell-specific promoters [105]. Limiting SSADH restoration to relevant cell populations is particularly important from a metabolic homeostasis standpoint, given that aberrant *aldh5a1* gene activities have been linked to oncogenesis [106,107].

This novel *aldh5a1^STOP/STOP^* mouse will be useful as a platform to develop other novel SSADH-targeted strategies. For example, *ALDH5A1* mutations in certain patients might be amenable via anti-sense oligonucleotide silencing [108] or frameshift [109]. Small molecules such as mRNA or recombinant enzymes might be encapsulated by lipid nanoparticles for targeted delivery [110]. In addition, mouse-derived induced pluripotent stem cells (iPSCs) might provide mechanistic insights into therapeutic SSADH restoration in vitro. Future experiments using patient-derived iPSCs might be developed into a novel cell-based therapy.

## 6. Concluding Remarks

SSADHD research has come a long way since the initial clinical documentation and biochemical discovery. A great deal of knowledge has been accumulated regarding the molecular, metabolic, and neurochemical underpinnings of this rare genetic disorder. Multiple symptom-targeting drugs have been trialed, but results suggest that their therapeutic utility is rather limited. SSADH-targeted medicine utilizing gene therapy and ERT technology is a likely promising approach, but we are cautious about potential risks if administered improperly. Therefore, we propose to use a novel SSADHD mouse model to de-risk SSADH restoration strategies. We envision that this work in conjunction with the ongoing SSADHD Natural History Study will together accelerate the bench to bedside development of a cure for SSADHD.

## 7. Materials and Methods

### 7.1. Animal Use and Institutional Assurance

All animal housing and breeding procedures performed in this study were covered by protocols approved by the Institutional Animal Care and Use Committee at Boston Children’s Hospital and in accordance with the National Institutes of Health (NIH) Guide for the Care and Use of Laboratory Animals. Mice were housed in standard cages in a temperature-controlled facility with 12 h light/dark cycle and continuous supply of water and food ad libitum. Mouse survival was checked daily from birth to postnatal days 100. Both male and female mice were used in this study and in the data presented.

### 7.2. Western Blot Analyses

Mice were sacrificed under acute isoflurane anesthesia. After decapitation, brain tissue was quickly removed and micro-dissected on an ice-cold platform into cortex, hippocampus, and cerebellum, followed by snap freezing using liquid nitrogen. Protein content of frozen cortical tissues was extracted using T-PER Tissue Protein Extraction Reagent (Thermo Scientific, Waltham, MA, USA) assisted with ultrasonic disruption and homogenization. Undissolved materials were removed using benchtop microcentrifuge (4 °C, 14,000 rpm, 20 min). Sample protein contents were measured by BCA protein assay (Thermo Scientific, Waltham, MA, USA), resolved by polyacrylamide gel electrophoresis (PAGE) using Criterion TGX 4–20% Precast Gels (Biorad, Hercules, CA, USA), and transferred onto PVDF membrane using the iBlot gel transfer system (Invitrogen, Waltham, MA, USA). Membrane was blocked for 1 h at room temperature using Intercept (TBS) blocking buffer (Licor, Lincoln, NE, USA), incubated overnight at 4 °C with primary antibodies (rabbit anti-SSADH, mouse anti-β-actin, Abcam, Cambridge, UK), washed by TBS-T, incubated for 1 h at room temperature with IRDye 800 CW anti-rabbit and 680 RD anti-mouse secondary antibodies (Licor, Lincoln, NE, USA), and imaged using Odyssey imaging system (Licor, Lincoln, NE, USA). Images were processed and quantified using Image Studio acquisition software (Licor, Lincoln, NE, USA).

### 7.3. Data Processing and Statistical Analysis

Western blot analyses were carried out by measuring and comparing protein band intensity. SSADH amount in each sample is normalized by its own β-actin amount, then calculated compared to WT control levels. All data are included and statistically tested using unpaired t-test in GraphPad Prism, represented as means ± standard error of the mean.

## Data Availability

Data is available upon request.

## References

[1] Jakobs C., Bojasch M., Monch E., Rating D., Siemes H., Hanefeld F. (1981). Urinary excretion of gamma-hydroxybutyric acid in a patient with neurological abnormalities. The probability of a new inborn error of metabolism. Clin. Chim. Acta.

[2] Gibson K.M., Sweetman L., Nyhan W.L., Jakobs C., Rating D., Siemes H., Hanefeld F. (1983). Succinic semialdehyde dehydrogenase deficiency: An inborn error of gamma-aminobutyric acid metabolism. Clin. Chim. Acta.

[3] Gibson K.M., Baumann C., Ogier H., Rossier E., Vollmer B., Jakobs C. (1994). Pre- and postnatal diagnosis of succinic semialdehyde dehydrogenase deficiency using enzyme and metabolite assays. J. Inherit. Metab. Dis..

[4] Bernocchi G., Barni S., Biggiogera M. (1986). Electron-cytochemical localization of succinic semialdehyde dehydrogenase activity in Purkinje neurons and hepatocytes of the rat. J. Neurosci. Methods.

[5] Chambliss K.L., Caudle D.L., Hinson D.D., Moomaw C.R., Slaughter C.A., Jakobs C., Gibson K.M. (1995). Molecular cloning of the mature NAD(+)-dependent succinic semialdehyde dehydrogenase from rat and human. cDNA isolation, evolutionary homology, and tissue expression. J. Biol. Chem..

[6] Aoshima T., Kajita M., Sekido Y., Ishiguro Y., Tsuge I., Kimura M., Yamaguchi S., Watanabe K., Shimokata K., Niwa T. (2002). Mutation analysis in a patient with succinic semialdehyde dehydrogenase deficiency: A compound heterozygote with 103-121del and 1460T > A of the ALDH5A1 gene. Hum. Hered..

[7] Akaboshi S., Hogema B.M., Novelletto A., Malaspina P., Salomons G.S., Maropoulos G.D., Jakobs C., Grompe M., Gibson K.M. (2003). Mutational spectrum of the succinate semialdehyde dehydrogenase (ALDH5A1) gene and functional analysis of 27 novel disease-causing mutations in patients with SSADH deficiency. Hum. Mutat..

[8] Liu N., Kong X.D., Kan Q.C., Shi H.R., Wu Q.H., Zhuo Z.H., Bai Q.L., Jiang M. (2016). Mutation analysis and prenatal diagnosis in a Chinese family with succinic semialdehyde dehydrogenase and a systematic review of the literature of reported ALDH5A1 mutations. J. Perinat. Med..

[9] Leo S., Capo C., Ciminelli B.M., Iacovelli F., Menduti G., Funghini S., Donati M.A., Falconi M., Rossi L., Malaspina P. (2017). SSADH deficiency in an Italian family: A novel ALDH5A1 gene mutation affecting the succinic semialdehyde substrate binding site. Metab. Brain Dis..

[10] Chen X.D., Lin Y.T., Jiang M.Y., Li X.Z., Li D., Hu H., Liu L. (2020). Novel mutations in a Chinese family with two patients with succinic semialdehyde dehydrogenase deficiency. Gynecol. Endocrinol..

[11] DiBacco M.L., Pop A., Salomons G.S., Hanson E., Roullet J.B., Gibson K.M., Pearl P.L. (2020). Novel ALDH5A1 variants and genotype: Phenotype correlation in SSADH deficiency. Neurology.

[12] Pearl P.L., Wiwattanadittakul N., Roullet J.B., Gibson K.M., Adam M.P., Ardinger H.H., Pagon R.A., Wallace S.E., Bean L.J.H., Stephens K., Amemiya A. (1993). Succinic Semialdehyde Dehydrogenase Deficiency. GeneReviews^®^.

[13] Gibson K.M., Christensen E., Jakobs C., Fowler B., Clarke M.A., Hammersen G., Raab K., Kobori J., Moosa A., Vollmer B. (1997). The clinical phenotype of succinic semialdehyde dehydrogenase deficiency (4-hydroxybutyric aciduria): Case reports of 23 new patients. Pediatrics.

[14] Pearl P.L., Gibson K.M., Acosta M.T., Vezina L.G., Theodore W.H., Rogawski M.A., Novotny E.J., Gropman A., Conry J.A., Berry G.T. (2003). Clinical spectrum of succinic semialdehyde dehydrogenase deficiency. Neurology.

[15] Knerr I., Gibson K.M., Jakobs C., Pearl P.L. (2008). Neuropsychiatric morbidity in adolescent and adult succinic semialdehyde dehydrogenase deficiency patients. CNS Spectr..

[16] Gibson K.M., Gupta M., Pearl P.L., Tuchman M., Vezina L.G., Snead O.C., Smit L.M., Jakobs C. (2003). Significant behavioral disturbances in succinic semialdehyde dehydrogenase (SSADH) deficiency (gamma-hydroxybutyric aciduria). Biol. Psychiatry.

[17] Pearl P.L., Shukla L., Theodore W.H., Jakobs C., Michael Gibson K. (2011). Epilepsy in succinic semialdehyde dehydrogenase deficiency, a disorder of GABA metabolism. Brain Dev..

[18] DiBacco M.L., Roullet J.B., Kapur K., Brown M.N., Walters D.C., Gibson K.M., Pearl P.L. (2019). Age-related phenotype and biomarker changes in SSADH deficiency. Ann. Clin. Transl. Neurol..

[19] Walters D.C., Lawrence R., Kirby T., Ahrendsen J.T., Anderson M.P., Roullet J.B., Murphy E.J., Gibson K.M., Consortium S.D.I. (2021). Postmortem Analyses in a Patient With Succinic Semialdehyde Dehydrogenase Deficiency (SSADHD): II. Histological, Lipid, and Gene Expression Outcomes in Regional Brain Tissue. J. Child Neurol..

[20] Hogema B.M., Akaboshi S., Taylor M., Salomons G.S., Jakobs C., Schutgens R.B., Wilcken B., Worthington S., Maropoulos G., Grompe M. (2001). Prenatal diagnosis of succinic semialdehyde dehydrogenase deficiency: Increased accuracy employing DNA, enzyme, and metabolite analyses. Mol. Genet. Metab..

[21] Gropman A. (2003). Vigabatrin and newer interventions in succinic semialdehyde dehydrogenase deficiency. Ann. Neurol..

[22] Ergezinger K., Jeschke R., Frauendienst-Egger G., Korall H., Gibson K.M., Schuster V.H. (2003). Monitoring of 4-hydroxybutyric acid levels in body fluids during vigabatrin treatment in succinic semialdehyde dehydrogenase deficiency. Ann. Neurol..

[23] Matern D., Lehnert W., Gibson K.M., Korinthenberg R. (1996). Seizures in a boy with succinic semialdehyde dehydrogenase deficiency treated with vigabatrin (gamma-vinyl-GABA). J. Inherit. Metab. Dis..

[24] Vanadia E., Gibson K.M., Pearl P.L., Trapolino E., Mangano S., Vanadia F. (2013). Therapeutic efficacy of magnesium valproate in succinic semialdehyde dehydrogenase deficiency. JIMD Rep..

[25] Kratz S.V. (2009). Sensory integration intervention: Historical concepts, treatment strategies and clinical experiences in three patients with succinic semialdehyde dehydrogenase (SSADH) deficiency. J. Inherit. Metab. Dis..

[26] Vogel K.R., Pearl P.L., Theodore W.H., McCarter R.C., Jakobs C., Gibson K.M. (2013). Thirty years beyond discovery--clinical trials in succinic semialdehyde dehydrogenase deficiency, a disorder of GABA metabolism. J. Inherit. Metab. Dis..

[27] Hogema B.M., Gupta M., Senephansiri H., Burlingame T.G., Taylor M., Jakobs C., Schutgens R.B., Froestl W., Snead O.C., Diaz-Arrastia R. (2001). Pharmacologic rescue of lethal seizures in mice deficient in succinate semialdehyde dehydrogenase. Nat. Genet..

[28] Cortez M.A., Wu Y., Gibson K.M., Snead O.C. (2004). Absence seizures in succinic semialdehyde dehydrogenase deficient mice: A model of juvenile absence epilepsy. Pharmacol. Biochem. Behav..

[29] Gibson K.M., Jakobs C., Pearl P.L., Snead O.C. (2005). Murine succinate semialdehyde dehydrogenase (SSADH) deficiency, a heritable disorder of GABA metabolism with epileptic phenotype. IUBMB Life.

[30] Stewart L.S., Nylen K.J., Persinger M.A., Cortez M.A., Gibson K.M., Snead O.C. (2008). Circadian distribution of generalized tonic-clonic seizures associated with murine succinic semialdehyde dehydrogenase deficiency, a disorder of GABA metabolism. Epilepsy Behav..

[31] Gupta M., Greven R., Jansen E.E., Jakobs C., Hogema B.M., Froestl W., Snead O.C., Bartels H., Grompe M., Gibson K.M. (2002). Therapeutic intervention in mice deficient for succinate semialdehyde dehydrogenase (gamma-hydroxybutyric aciduria). J. Pharmacol. Exp. Ther..

[32] Vogel K.R., Ainslie G.R., McConnell A., Roullet J.B., Gibson K.M. (2018). Toxicologic/transport properties of NCS-382, a gamma-hydroxybutyrate (GHB) receptor ligand, in neuronal and epithelial cells: Therapeutic implications for SSADH deficiency, a GABA metabolic disorder. Toxicol. In Vitr..

[33] Vogel K.R., Ainslie G.R., Gibson K.M. (2016). mTOR inhibitors rescue premature lethality and attenuate dysregulation of GABAergic/glutamatergic transcription in murine succinate semialdehyde dehydrogenase deficiency (SSADHD), a disorder of GABA metabolism. J. Inherit. Metab. Dis..

[34] Vogel K.R., Ainslie G.R., Jansen E.E., Salomons G.S., Gibson K.M. (2015). Torin 1 partially corrects vigabatrin-induced mitochondrial increase in mouse. Ann. Clin. Transl. Neurol..

[35] Nylen K., Velazquez J.L., Likhodii S.S., Cortez M.A., Shen L., Leshchenko Y., Adeli K., Gibson K.M., Burnham W.M., Snead O.C. (2008). A ketogenic diet rescues the murine succinic semialdehyde dehydrogenase deficient phenotype. Exp. Neurol..

[36] Nylen K., Velazquez J.L., Sayed V., Gibson K.M., Burnham W.M., Snead O.C. (2009). The effects of a ketogenic diet on ATP concentrations and the number of hippocampal mitochondria in Aldh5a1(−/−) mice. Biochim. Biophys. Acta.

[37] Pearl P.L., Schreiber J., Theodore W.H., McCarter R., Barrios E.S., Yu J., Wiggs E., He J., Gibson K.M. (2014). Taurine trial in succinic semialdehyde dehydrogenase deficiency and elevated CNS GABA. Neurology.

[38] Schreiber J.M., Pearl P.L., Dustin I., Wiggs E., Barrios E., Wassermann E.M., Gibson K.M., Theodore W.H. (2016). Biomarkers in a Taurine Trial for Succinic Semialdehyde Dehydrogenase Deficiency. JIMD Rep..

[39] Schreiber J.M., Wiggs E., Cuento R., Norato G., Dustin I.H., Rolinski R., Austermuehle A., Zhou X., Inati S.K., Gibson K.M. (2021). A Randomized Controlled Trial of SGS-742, a gamma-aminobutyric acid B (GABA-B) Receptor Antagonist, for Succinic Semialdehyde Dehydrogenase Deficiency. J. Child Neurol..

[40] Gupta M., Jansen E.E., Senephansiri H., Jakobs C., Snead O.C., Grompe M., Gibson K.M. (2004). Liver-directed adenoviral gene transfer in murine succinate semialdehyde dehydrogenase deficiency. Mol. Ther..

[41] Vogel K.R., Ainslie G.R., Walters D.C., McConnell A., Dhamne S.C., Rotenberg A., Roullet J.B., Gibson K.M. (2018). Succinic semialdehyde dehydrogenase deficiency, a disorder of GABA metabolism: An update on pharmacological and enzyme-replacement therapeutic strategies. J. Inherit. Metab. Dis..

[42] Bulaklak K., Gersbach C.A. (2020). The once and future gene therapy. Nat. Commun..

[43] Fischer J.S. (2000). Best hope or broken promise? After a decade, gene therapy goes on trial. US News World Rep..

[44] Papanikolaou E., Bosio A. (2021). The Promise and the Hope of Gene Therapy. Front. Genome Ed..

[45] Connock M., Juarez-Garcia A., Frew E., Mans A., Dretzke J., Fry-Smith A., Moore D. (2006). A systematic review of the clinical effectiveness and cost-effectiveness of enzyme replacement therapies for Fabry’s disease and mucopolysaccharidosis type 1. Health Technol. Assess..

[46] Safary A., Akbarzadeh Khiavi M., Mousavi R., Barar J., Rafi M.A. (2018). Enzyme replacement therapies: What is the best option?. Bioimpacts.

[47] Pearl P.L., DiBacco M.L., Papadelis C., Opladen T., Hanson E., Roullet J.B., Gibson K.M. (2021). Succinic Semialdehyde Dehydrogenase Deficiency: Review of the Natural History Study. J. Child Neurol..

[48] Lee H.H.C., Pearl P.L., Rotenberg A. (2021). Enzyme Replacement Therapy for Succinic Semialdehyde Dehydrogenase Deficiency: Relevance in gamma-Aminobutyric Acid Plasticity. J. Child Neurol..

[49] Prevot T., Sibille E. (2021). Altered GABA-mediated information processing and cognitive dysfunctions in depression and other brain disorders. Mol. Psychiatry.

[50] Chiu C.Q., Barberis A., Higley M.J. (2019). Preserving the balance: Diverse forms of long-term GABAergic synaptic plasticity. Nat. Rev. Neurosci..

[51] Madsen K.K., Larsson O.M., Schousboe A. (2008). Regulation of excitation by GABA neurotransmission: Focus on metabolism and transport. Results Probl. Cell Differ..

[52] Mohler H. (2006). GABA(A) receptor diversity and pharmacology. Cell Tissue Res..

[53] Terunuma M. (2018). Diversity of structure and function of GABAB receptors: A complexity of GABAB-mediated signaling. Proc. Jpn. Acad. Ser. B Phys. Biol. Sci..

[54] Jacob T.C., Moss S.J., Jurd R. (2008). GABA(A) receptor trafficking and its role in the dynamic modulation of neuronal inhibition. Nat. Rev. Neurosci..

[55] Tritsch N.X., Granger A.J., Sabatini B.L. (2016). Mechanisms and functions of GABA co-release. Nat. Rev. Neurosci..

[56] Brickley S.G., Mody I. (2012). Extrasynaptic GABA(A) receptors: Their function in the CNS and implications for disease. Neuron.

[57] Field M., Dorovykh V., Thomas P., Smart T.G. (2021). Physiological role for GABAA receptor desensitization in the induction of long-term potentiation at inhibitory synapses. Nat. Commun..

[58] Brooks-Kayal A.R., Russek S.J., Noebels J.L., Avoli M., Rogawski M.A., Olsen R.W., Delgado-Escueta A.V. (2012). Regulation of GABAA Receptor Gene Expression and Epilepsy. Jasper’s Basic Mechanisms of the Epilepsies.

[59] Gassmann M., Bettler B. (2012). Regulation of neuronal GABA(B) receptor functions by subunit composition. Nat. Rev. Neurosci..

[60] Mele M., Costa R.O., Duarte C.B. (2019). Alterations in GABAA-Receptor Trafficking and Synaptic Dysfunction in Brain Disorders. Front. Cell Neurosci..

[61] Wong C.G., Bottiglieri T., Snead O.C. (2003). GABA, gamma-hydroxybutyric acid, and neurological disease. Ann. Neurol..

[62] Jansen E.E., Vogel K.R., Salomons G.S., Pearl P.L., Roullet J.B., Gibson K.M. (2016). Correlation of blood biomarkers with age informs pathomechanisms in succinic semialdehyde dehydrogenase deficiency (SSADHD), a disorder of GABA metabolism. J. Inherit. Metab. Dis..

[63] Afacan O., Yang E., Lin A.P., Coello E., DiBacco M.L., Pearl P.L., Warfield S.K., Consortium S.D.I. (2021). Magnetic Resonance Imaging (MRI) and Spectroscopy in Succinic Semialdehyde Dehydrogenase Deficiency. J. Child Neurol..

[64] Tsuboyama M., Liu J., Kaye H., DiBacco M., Pearl P.L., Rotenberg A. (2021). Transcranial Magnetic Stimulation in Succinic Semialdehyde Dehydrogenase Deficiency: A Measure of Maturational Trajectory of Cortical Excitability. J. Child Neurol..

[65] Pearl P.L., Gibson K.M., Quezado Z., Dustin I., Taylor J., Trzcinski S., Schreiber J., Forester K., Reeves-Tyer P., Liew C. (2009). Decreased GABA-A binding on FMZ-PET in succinic semialdehyde dehydrogenase deficiency. Neurology.

[66] Ben-Ari Y., Khalilov I., Kahle K.T., Cherubini E. (2012). The GABA excitatory/inhibitory shift in brain maturation and neurological disorders. Neuroscientist.

[67] Lee H.H., Deeb T.Z., Walker J.A., Davies P.A., Moss S.J. (2011). NMDA receptor activity downregulates KCC2 resulting in depolarizing GABAA receptor-mediated currents. Nat. Neurosci..

[68] Brennan R., Van Hout M.C. (2014). Gamma-hydroxybutyrate (GHB): A scoping review of pharmacology, toxicology, motives for use, and user groups. J. Psychoact. Drugs.

[69] Xu X.M., Wei Y.D., Liu Y., Li Z.X. (2019). Gamma-hydroxybutyrate (GHB) for narcolepsy in adults: An updated systematic review and meta-analysis. Sleep Med..

[70] Maitre M. (1997). The gamma-hydroxybutyrate signalling system in brain: Organization and functional implications. Prog. Neurobiol..

[71] Gibson K.M., Hoffmann G.F., Hodson A.K., Bottiglieri T., Jakobs C. (1998). 4-Hydroxybutyric acid and the clinical phenotype of succinic semialdehyde dehydrogenase deficiency, an inborn error of GABA metabolism. Neuropediatrics.

[72] Andriamampandry C., Taleb O., Viry S., Muller C., Humbert J.P., Gobaille S., Aunis D., Maitre M. (2003). Cloning and characterization of a rat brain receptor that binds the endogenous neuromodulator gamma-hydroxybutyrate (GHB). FASEB J..

[73] Absalom N., Eghorn L.F., Villumsen I.S., Karim N., Bay T., Olsen J.V., Knudsen G.M., Brauner-Osborne H., Frolund B., Clausen R.P. (2012). alpha4betadelta GABA(A) receptors are high-affinity targets for gamma-hydroxybutyric acid (GHB). Proc. Natl. Acad. Sci. USA.

[74] Molnar T., Antal K., Nyitrai G., Emri Z. (2009). gamma-Hydroxybutyrate (GHB) induces GABA(B) receptor independent intracellular Ca^2+^ transients in astrocytes, but has no effect on GHB or GABA(B) receptors of medium spiny neurons in the nucleus accumbens. Neuroscience.

[75] Bay T., Eghorn L.F., Klein A.B., Wellendorph P. (2014). GHB receptor targets in the CNS: Focus on high-affinity binding sites. Biochem. Pharmacol..

[76] Snead O.C. (2000). Evidence for a G protein-coupled gamma-hydroxybutyric acid receptor. J. Neurochem..

[77] Banerjee P.K., Snead O.C. (1995). Presynaptic gamma-hydroxybutyric acid (GHB) and gamma-aminobutyric acidB (GABAB) receptor-mediated release of GABA and glutamate (GLU) in rat thalamic ventrobasal nucleus (VB): A possible mechanism for the generation of absence-like seizures induced by GHB. J. Pharmacol. Exp. Ther..

[78] Howard S.G., Feigenbaum J.J. (1997). Effect of gamma-hydroxybutyrate on central dopamine release in vivo. A microdialysis study in awake and anesthetized animals. Biochem. Pharmacol..

[79] Feigenbaum J.J., Howard S.G. (1996). Does gamma-hydroxybutyrate inhibit or stimulate central DA release?. Int. J. Neurosci..

[80] Allen N.J., Eroglu C. (2017). Cell Biology of Astrocyte-Synapse Interactions. Neuron.

[81] Pajarillo E., Rizor A., Lee J., Aschner M., Lee E. (2019). The role of astrocytic glutamate transporters GLT-1 and GLAST in neurological disorders: Potential targets for neurotherapeutics. Neuropharmacology.

[82] Schousboe A., Bak L.K., Waagepetersen H.S. (2013). Astrocytic Control of Biosynthesis and Turnover of the Neurotransmitters Glutamate and GABA. Front. Endocrinol..

[83] Rigby M.J., Gomez T.M., Puglielli L. (2020). Glial Cell-Axonal Growth Cone Interactions in Neurodevelopment and Regeneration. Front. Neurosci..

[84] Abbott N.J., Ronnback L., Hansson E. (2006). Astrocyte-endothelial interactions at the blood-brain barrier. Nat. Rev. Neurosci..

[85] Li S., Kumar T.P., Joshee S., Kirschestein T., Subburaju S., Khalili J.S., Du C., Elkhal A., Szabó G., Jain R.K. (2018). Endothelial cell-derived GABA signaling modulates neuronal migration and postnatal behavior. Cell Res..

[86] Verhoog Q.P., Holtman L., Aronica E., van Vliet E.A. (2020). Astrocytes as Guardians of Neuronal Excitability: Mechanisms Underlying Epileptogenesis. Front. Neurol..

[87] Coulter D.A., Steinhauser C. (2015). Role of astrocytes in epilepsy. Cold Spring Harb. Perspect. Med..

[88] Xing L., Yang T., Cui S., Chen G. (2019). Connexin Hemichannels in Astrocytes: Role in CNS Disorders. Front. Mol. Neurosci..

[89] Goodenough D.A., Paul D.L. (2009). Gap junctions. Cold Spring Harb. Perspect. Biol..

[90] Froes M.M., Correia A.H., Garcia-Abreu J., Spray D.C., Campos de Carvalho A.C., Neto M.V. (1999). Gap-junctional coupling between neurons and astrocytes in primary central nervous system cultures. Proc. Natl. Acad. Sci. USA.

[91] Wallraff A., Kohling R., Heinemann U., Theis M., Willecke K., Steinhauser C. (2006). The impact of astrocytic gap junctional coupling on potassium buffering in the hippocampus. J. Neurosci..

[92] Takahashi D.K., Vargas J.R., Wilcox K.S. (2010). Increased coupling and altered glutamate transport currents in astrocytes following kainic-acid-induced status epilepticus. Neurobiol. Dis..

[93] Bennett M.V., Contreras J.E., Bukauskas F.F., Saez J.C. (2003). New roles for astrocytes: Gap junction hemichannels have something to communicate. Trends Neurosci..

[94] Kuhn S., Gritti L., Crooks D., Dombrowski Y. (2019). Oligodendrocytes in Development, Myelin Generation and Beyond. Cells.

[95] Benamer N., Vidal M., Balia M., Angulo M.C. (2020). Myelination of parvalbumin interneurons shapes the function of cortical sensory inhibitory circuits. Nat. Commun..

[96] Hu X., Wang J.Y., Gu R., Qu H., Li M., Chen L., Liu R., Yuan P. (2016). The relationship between the occurrence of intractable epilepsy with glial cells and myelin sheath—An experimental study. Eur Rev. Med. Pharmacol. Sci..

[97] Nualart-Marti A., Solsona C., Fields R.D. (2013). Gap junction communication in myelinating glia. Biochim. Biophys. Acta.

[98] Chowdhury G.M., Gupta M., Gibson K.M., Patel A.B., Behar K.L. (2007). Altered cerebral glucose and acetate metabolism in succinic semialdehyde dehydrogenase-deficient mice: Evidence for glial dysfunction and reduced glutamate/glutamine cycling. J. Neurochem..

[99] Donarum E.A., Stephan D.A., Larkin K., Murphy E.J., Gupta M., Senephansiri H., Switzer R.C., Pearl P.L., Snead O.C., Jakobs C. (2006). Expression profiling reveals multiple myelin alterations in murine succinate semialdehyde dehydrogenase deficiency. J. Inherit. Metab. Dis..

[100] Barcelo-Coblijn G., Murphy E.J., Mills K., Winchester B., Jakobs C., Snead O.C., Gibson K.M. (2007). Lipid abnormalities in succinate semialdehyde dehydrogenase (Aldh5a1−/-/-) −) deficient mouse brain provide additional evidence for myelin alterations. Biochim. Biophys. Acta.

[101] Gupta M., Polinsky M., Senephansiri H., Snead O.C., Jansen E.E., Jakobs C., Gibson K.M. (2004). Seizure evolution and amino acid imbalances in murine succinate semialdehyde dehydrogenase (SSADH) deficiency. Neurobiol. Dis..

[102] Taniguchi H., He M., Wu P., Kim S., Paik R., Sugino K., Kvitsiani D., Fu Y., Lu J., Lin Y. (2011). A resource of Cre driver lines for genetic targeting of GABAergic neurons in cerebral cortex. Neuron.

[103] Gorski J.A., Talley T., Qiu M., Puelles L., Rubenstein J.L., Jones K.R. (2002). Cortical excitatory neurons and glia, but not GABAergic neurons, are produced in the Emx1-expressing lineage. J. Neurosci..

[104] Gregorian C., Nakashima J., Le Belle J., Ohab J., Kim R., Liu A., Smith K.B., Groszer M., Garcia A.D., Sofroniew M.V. (2009). Pten deletion in adult neural stem/progenitor cells enhances constitutive neurogenesis. J. Neurosci..

[105] Liu Y., Hegarty S., Winter C., Wang F., He Z. (2020). Viral vectors for neuronal cell type-specific visualization and manipulations. Curr. Opin. Neurobiol..

[106] Deng X.Y., Gan X.X., Feng J.H., Cai W.S., Wang X.Q., Shen L., Luo H.T., Chen Z., Guo M., Cao J. (2021). ALDH5A1 acts as a tumour promoter and has a prognostic impact in papillary thyroid carcinoma. Cell Biochem. Funct..

[107] Hujber Z., Horvath G., Petovari G., Krencz I., Danko T., Meszaros K., Rajnai H., Szoboszlai N., Leenders W.P.J., Jeney A. (2018). GABA, glutamine, glutamate oxidation and succinic semialdehyde dehydrogenase expression in human gliomas. J. Exp. Clin. Cancer Res..

[108] Watts J.K., Corey D.R. (2012). Silencing disease genes in the laboratory and the clinic. J. Pathol..

[109] Henderson C.M., Anderson C.B., Howard M.T. (2006). Antisense-induced ribosomal frameshifting. Nucleic Acids Res..

[110] Scioli Montoto S., Muraca G., Ruiz M.E. (2020). Solid Lipid Nanoparticles for Drug Delivery: Pharmacological and Biopharmaceutical Aspects. Front. Mol. Biosci..

